# Utility of the modified ATP III defined metabolic syndrome and severe obesity as predictors of insulin resistance in overweight children and adolescents: a cross-sectional study

**DOI:** 10.1186/1475-2840-6-4

**Published:** 2007-02-14

**Authors:** Sarita Dhuper, Hillel W Cohen, Josephine Daniel, Padmasree Gumidyala, Vipin Agarwalla, Rosemarie St Victor, Sunil Dhuper

**Affiliations:** 1 Department of Pediatrics, Brookdale University Hospital & Medical Center, Brooklyn, NY, USA; 2 Department of Epidemiology and Population Health, Albert Einstein College of Medicine, Bronx, NY, USA; 3 Department of Medicine, Coney Island Hospital, Brooklyn, New York, USA

## Abstract

**Background:**

The rising prevalence of obesity and metabolic syndrome (MetS) has received increased attention since both place individuals at risk for Type II diabetes and cardiovascular disease. Insulin resistance (IR) has been implicated in the pathogenesis of obesity and MetS in both children and adults and is a known independent cardiovascular risk factor. However measures of IR are not routinely performed in children while MetS or severe obesity when present, are considered as clinical markers for IR.

**Objective:**

The study was undertaken to assess the utility of ATPIII defined metabolic syndrome (MetS) and severe obesity as predictors of insulin resistance (IR) in a group of 576 overweight children and adolescents attending a pediatric obesity clinic in Brooklyn.

**Methods:**

Inclusion criteria were children ages 3–19, and body mass index > 95th percentile for age. MetS was defined using ATP III criteria, modified for age. IR was defined as upper tertile of homeostasis model assessment (HOMA) within 3 age groups (3–8, n = 122; 9–11, n = 164; 12–19, n = 290). Sensitivity, specificity, positive predictive values and odds ratios (OR) with 95% confidence intervals (CI) were calculated within age groups for predicting IR using MetS and severe obesity respectively.

**Results:**

MetS was present in 45%, 48% and 42% of the respective age groups and significantly predicted IR only in the oldest group (OR = 2.0, 95% CI 1.2, 3.4; p = .006). Sensitivities were <55%; specificities <63% and positive predictive values ≤ 42% in all groups. Severe obesity was significantly associated with IR in both the 9–11 (p = .002) and 12–18 (p = .01) groups but positive predictive values were nonetheless ≤ 51% for all groups.

**Conclusion:**

The expression of IR in overweight children and adolescents is heterogeneous and MetS or severe obesity may not be sufficiently sensitive and specific indicators of insulin resistance. In addition to screening for MetS in overweight children markers for IR should be routinely performed. Further research is needed to establish threshold values of insulin measures in overweight children who may be at greater associated risk of adverse outcomes whether or not MetS is present.

## Background

Insulin resistance (IR) and/or compensatory hyperinsulinemia are usually associated with obesity and are risk factors for cardiovascular disease (CVD) and type 2 diabetes in both adults and children [1-8]. Furthermore, it is now clear that polycystic ovary syndrome [9] nonalcoholic liver disease [10] sleep disordered breathing [11], renal disease, systemic inflammation, [12,13] asthma [14] and several types of cancer [15] are also associated with IR. Given the increasing prevalence of obesity, type 2 diabetes [16] and metabolic syndrome (MetS) in children[17], and the known relationship of IR as a precursor to these clinical syndromes [1-3], identifying IR in children may be of substantial clinical importance. Although MetS and IR undoubtedly overlap, they are not the same [18,19]. The NCEP ATP III. (National Cholesterol Education Program, Adult treatment panel III) definition of MetS [20] does not include a direct measure of insulin and it is likely that IR confers CVD or other disease risk distinct from that conferred by MetS itself [21-25]. Recent longitudinal studies in children have shown that cardiovascular risk factors like blood pressure and triglycerides decreased with any degree of decrease in HOMA, independent of changes in weight status, supporting the hypothesis that insulin resistance is a key abnormality contributing to these cardiovascular risk factors [26]. In light of the substantial overlap of MetS and IR, the question arises whether a direct measure of insulin or glucose tolerance is needed when MetS is more easily assessed and could be used as a marker for IR. Recently, however, the clinical utility of MetS to serve as a marker of IR has been questioned in adults [27,28]. We have undertaken the current study to examine this question in a population of overweight children.

## Methods

Data were collected on all overweight children and adolescents(3–19 years) with BMI > 95th percentile for age, (BMIZ range 1.2, 6.3) who attended a pediatric obesity program at an inner city University Hospital, in Brooklyn, New York from January1, 2002 through December 31, 2005. Five hundred and seventy six (576) patients with complete data for metabolic risk factors constituted the study sample. The study was approved by the Internal Review Board and informed consent to participate in the community based obesity program and to be included in the database was obtained from the parent or guardian and assent from the children when first enrolled. Each individual was assessed for the presence of MetS. Fasting plasma concentrations of glucose, insulin, triglycerides, and HDL cholesterol were assessed using standard laboratory methods. Weight and height were measured to the nearest 0.1 kg and 0.5 cm respectively. BMI was calculated as weight in kilograms divided by height in meters squared and converted to BMI-Z score standard units according to CDC age-sex tables [29]. Waist circumference was measured at the midpoint between the lateral iliac crest and the lowest rib in cm during expiration and defined as abnormal if > 90th percentile for age, sex and race [30]. Blood pressure was measured with an appropriate size cuff with a standardized automated dynamapp in the right arm in the sitting position and the average of three readings was recorded for analysis. Elevated systolic or diastolic blood pressure was defined as values above the 90th percentile for age and gender [31]. Triglycerides were characterized as elevated if ≥ 90th percentile for age and sex based on national standards [32]. HDL ≤ 40 mg/dL were considered abnormal [32]. Fasting glucose ≥ 100 mg/dL was considered elevated based on the revised American Diabetes Association criterion [33]. MetS was defined consistent with Cook et al.[17] as age-modified ATPIII criteria with abnormal values for at least 3 of the 5 criteria: systolic or diastolic blood pressure, fasting glucose, HDL, waist circumference and triglycerides.

Since IR is affected by age and pubertal status, and tanner stages were not recorded on all patients in the database, the population was further divided into 3 age groups (3–8, 9–11, and 12–19) reflecting prepubertal, pubertal and post pubertal stages and also to assess the age related associations of MetS and IR. Currently there are no accepted values to define insulin resistance in normal and overweight children, so we used the upper tertile of homeostasis model assessment (HOMA: fasting serum insulin (μunits/ml) × fasting plasma glucose (mmol/l)/22.5) within age group to denote higher IR consistent with previous work in both adults [34] and children[35]. Analyses were repeated using upper quartile HOMA for IR to check if the results were threshold dependent.

### Statistical analysis

Sensitivity, specificity, and positive predictive value of using MetS to identify higher IR were computed. In addition, similar to Weiss et al[35] we divided the patients into severe obesity (BMI Z score ≥ 2.5) and moderate obesity (BMI Z Score < 2.5) and analyzed the sensitivity, specificity, positive and negative predictive value of severe obesity to identify higher IR in these age groups. The associations of the five components of MetS as categorical variables and associations with higher IR within the three age groups were assessed with chi-square. Associations of continuous MetS variables within age group were assessed with analysis of variance. Associations of these with continuous HOMA and with higher IR (1 = higher, 0 = lower) were assessed with non-parametric Spearman's correlation coefficient. Binary logistic regression models predicting higher IR with MetS and BMIZ separately while adjusting for sex, age and race were also constructed. All analyses were performed within age group using SPSS for Windows (version 13). A two-tailed alpha of .05 was used to denote statistical significance.

## Results

Of the 576 participants, 122 were ages 3–8, 164 were ages 9–11 and 290 were 12–19. Almost all were African American (81.4 %) or Hispanic (16.0%). Table 1 describes sample characteristics and metabolic risk factors according to age groups. These groups did not differ significantly by race/ethnicity or sex. Each of the MetS factors as continuous variables increased significantly with age (p < .01 for linear trend for all). Application of the modified ATP III criteria to these groups identified MetS in 45.1 % of the younger children, 48.8% of the 9–11 year olds and 42.4% of the adolescents. Although the prevalence of MetS was similar in these age groups, the distribution of MetS components all differed significantly.

**Table 1:** Demographic and Metabolic Syndrome Characteristics by Age Group

Characteristic*	Ages 3–8 n = 122	Age 9–11 n = 164	Ages 12–19 n = 290	Total n = 576	p value
Female (%)	55.7	53.0	53.1	53.6	.87
Ethnicity (%)					.92
African American	79.5	81.7	82.1	81.4	
Hispanic	17.2	15.2	15.9	16.0	
Other	3.3	3.0	2.1	2.6	
Body Mass Index (BMI) (kg/m^2^)	26.8 ± 4.7	31.0 ± 5.5	37.1 ± 7.2	33.2 ± 7.6	<.001
Age-sex standardized Body Mass Index	2.8 ± 0.71	2.4 ± 0.42	2.4 ± 0.36	2.5 ± 0.50	<.001
(BMIZ) (standardized units)					
Glucose ≥ 100 mg/dL (%)	2.5	4.3	3.8	3.6	.71
Elevated Waist Circumference^† ^(%)	94.3	95.1	85.9	90.3	.001
High Density Lipoprotein <40 mg/dL (%)	39.3	45.7	50.0	46.5	.14
Elevated Triglycerides^† ^(%)	34.4	47.0	29.0	35.2	.001
Hypertension^† ^(%)	64.8	53.7	59.0	58.7	.17
Metabolic syndrome^‡ ^(%)	45.1	48.8	42.4	44.6	.49
Glucose (mg/dL)	80 ± 10.5	83 ± 11.1	85 ± 20.3	83 ± 16.4	.02
Waist Circumference (cm)	80 ± 11.4	95 ± 12.5	108 ± 15.7	98 ± 17.7	<.001
High Density Lipoprotein (mg/dL)	46 ± 11.5	43 ± 10.5	41 ± 9.4	43 ± 10.3	<.001
Triglycerides (mg/dL)	83 ± 33.9	95 ± 41.4	98 ± 54.3	94 ± 47.4	.01
Blood pressure (mmHg)					
Systolic	114 ± 10.7	118 ± 9.4	125 ± 10.9	121 ± 11.4	<.001
Diastolic	65 ± 8.2	69 ± 8.2	73 ± 8.9	70 ± 9.2	<.001

* Values are presented as % for categorical variables and mean ± standard deviation for continuous variables. P values for comparison by age assessed by chi-square for categorical variables and analysis of variance for continuous variables. All continuous variables had p < .01 for linear trend.

^† ^≥ 90^th ^percentile for age and sex.

^‡ ^By modified ATP criteria: abnormal values for 3 or more of waist circumference, high density lipoprotein, triglycerides, hypertension and glucose.

Defining higher IR by upper age-specific tertiles of HOMA, threshold values of 2.31 for ages 3–8, 4.29 for those 9–11 and 4.26 for the 12–19 year olds were obtained. Upper tertiles of fasting insulin for these age groups were 11.8, 21.1 and 20.5 (units) respectively. Adolescents with MetS (Table 2) were twice as likely as those without to have higher IR (odds ratio (OR) 2.0, 95% CI 1.2, 3.4, p <.01) and MetS as a predictor of higher IR had a sensitivity of 54%, a specificity of 63% and a positive predictive value of 42%. For the two younger age groups, the odds ratios of MetS as a predictor of higher IR were lower, (ages 3–8: OR 1.3, 95% CI 0.6, 2.7, p = 0.70; ages 9–11: OR: 1.4, 95% CI 0.7, 2.7: p = 0.41). The sensitivities, specificities and positive predictive values for these age groups were comparable to the adolescents. Severe obesity was significantly associated with IR in both the 9–11 (p = .002) and 12–18 (p = .01) groups but positive predictive values were nonetheless ≤ 51% for all groups.(Table 2)

**Table 2:** Using Metabolic Syndrome or Severe Obesity to Predict Higher Insulin Resistance*

Predictor	**n**^†^	**OR (95% CI)**	**p**	**Sensitivity**^‡^	**Specificity**	**PPV**
	
**Metabolic Syndrome**						
Ages 3–8	55	1.3 (0.6, 2.7)	.70	49%	57%	36%
9–11	79	1.4 (0.7, 2.7)	.41	54%	55%	37%
12–19	123	2.0 (1.2, 3.4)	.006	54%	63%	42%
**Severe Obesity**						
Ages 3–8	80	0.9 (0.4, 1.9)	.88	63%	33%	33%
9–11	65	3.0 (1.5, 5.9)	.002	57%	69%	48%
12–19	116	2.0 (1.2, 3.3)	.01	51%	66%	51%

*Metabolic syndrome (MetS) by modified ATP III criteria; higher insulin resistance: upper tertile of homeostatis model (HOMA); severe obesity: BMI Z score ≥ 2.5.

^† ^n = number in age group with MetS or severe obesity respectively; OR (95%CI): Odds ratio and 95^% ^confidence interval.

^‡ ^Sensitivity: % of those with higher insulin resistance who have predictor. Specificity: % of those without predictor who do not have higher insulin resistance. Positive predictive value (PPV): % of those with predictor who have higher insulin resistance.

Examining male and female subgroups separately gave results similar to the group as a whole.

Table 3 shows the associations of the individual criteria of MetS with higher IR. In all age groups, as expected, elevated glucose was significantly associated with higher IR and this was the only factor associated with IR in the youngest group. In the 9–11 group, only WC among the other risk factors for MetS had a borderline significant association with higher IR. Among the adolescents, both elevated triglycerides and WC showed an association with higher IR, and lower HDL and elevated blood pressure showed trends towards associations but these were not statistically significant.

**Table 3:** Metabolic Syndrome criteria and Insulin Resistance

	**Age 3–8**		**Age 9–11**		**Age 12–19**	
	**Higher IR**		**Higher IR**		**Higher IR**	
MetS Criterion*	**Yes**	**No**	**Yes**	**No**	**Yes**	**No**
	**n = 41**	**n = 81**	**n = 54**	**n = 110**	**n = 96**	**n = 194**
Waist Circumference %	95	94 (p > .99)	100.	93 (p = .05)	94	82 (p = .01)
Blood Pressure %	66	64 (p > .99)	56	53 (p = .86)	65	56 (p = .21)
Glucose %	7	0 (p = .04)	11	1 (p = .01)	9	1 (p < .01)
Triglycerides %	37	33 (p = .88)	48	46 (p = .96)	41	23 (p < .01)
HDL %	44	37 (p = .59)	48	45 (p = .79)	56	47 (p = .17)

	**Age 3–8**		**Age 9–11**		**Age 12–19**	
**Number of Components**^‡^		**Higher IR**^†^		**Higher IR**		**Higher IR**
	**n**	**% (n)**	**n**	**% (n)**	**n**	**% (n)**

None	3	0 (0)	1	0 (0)	12	8 (1)
Any one	21	29 (6)	32	22 (7)	67	24 (16)
Any two	43	35 (15)	52	35 (18)	88	31 (27)
Any three	40	35 (14)	51	37 (19)	80	35 (28)
Any four	15	40 (6)	26	31 (8)	38	53 (20)
All five	0	-	2	100 (2)	5	80 (4)

* Metabolic syndrome (MetS) by modified ATP III criteria: any 3 or more of waist circumference, blood pressure and triglycerides ≥ 90^th ^percentile, HDL ≤ 40 mg/dL, Glucose ≥ 100 mg/dl. Higher insulin resistance (IR): upper tertile of HOMA. Values presented as %.

^† ^% of the number (n) within that age group who had insulin resistance (IR), defined as upper tertile of HOMA.

^‡^Test of association (Spearman's rho) of number of categories with higher IR within age group: age 3–8 (p = .34), age 9–11 (p = .18), age 12–19 (p < .01).

Although there was a cumulative effect on the prevalence of higher IR with the presence of increasing number of components of MetS in all age groups, this trend was statistically significant only among the adolescents. Among the patients with less than 3 metabolic risk factors (non MetS), there was a prevalence of higher IR in 31% of the 3–8 yr old, 29% in the 9–11 yr old and 20% in those 12–19 yr old.

Table 4 shows the correlations of HOMA as a continuous variable with the individual MetS criteria and BMIZ scores also as continuous variables. As expected, glucose correlated significantly with HOMA for all age groups. Among the youngest group, waist circumference, and diastolic BP were also significantly associated with HOMA. Waist circumference and BMI Z-score were significantly associated with HOMA in the 9–11 age group. Among the adolescents all of the criteria were significantly associated with HOMA except for diastolic BP which had a borderline significant association.

**Table 4:** Correlations of continuous HOMA with components of Metabolic Syndrome and BMIZ for the 3 age groups.

	Age 3–8	Age 9–11	Age 12–19
Criterion*	n = 92	n = 123	n = 219
Waist Circ	.48 (p < .01)	.17 (p = .03)	.30 (p < .01)
Systolic BP	.09 (p = .32)	.09 (p = .26)	.15 (p = .01)
Diastolic BP	.20 (p = .03)	-.01 (p = .87)	.11 (p = .07)
Glucose	.37 (p < .01)	.43 (p < .01)	.38 (p < .01)
Triglycerides	.08 (p = .37)	.13 (p = .11)	.28 (p < .01)
HDL	-.09 (p = .33)	-.05 (p = .54)	-.19 (p < .01)
BMIZ	-.05 (p = .60)	.26 (p < .01)	.27 (p < .01)

*Values are Spearman's rho correlation coefficient of component with continuous measure of HOMA and (p value tests for probability of rho = 0).

In binary logistic regression models adjusting for sex, age and race, we found MetS and BMIZ were not significant predictors of higher IR (p = .55 and p = .24 respectively) for 3–8 year olds. For 9–11 year olds, MetS showed a non-significant trend (p = .13) while BMIZ was a significant predictor (p < .01). Both MetS and BMIZ were significantly associated with higher IR for 12–19 year olds (p < .01 for each). In sensitivity analyses, using upper quartile of HOMA instead of upper tertile to indicate higher insulin resistance gave similar results.

## Discussion

The principle findings in our study were that the ATP III definition of MetS, as modified for the pediatric populations, and severe obesity are but modest predictors of IR, defined here as upper tertile of HOMA in this sample of overweight, children and adolescents. We found that although the prevalence of MetS was high and relatively similar in the three age groups, a statistically significant association with IR was evident only in adolescence. As described in table 2 using MetS would miss 51%, 46% and 46% of those with IR for ages 3–8, 9–11 and 12–19 respectively and incorrectly label 43%, 45%, and 37% respectively as IR despite HOMA being in the lower two tertiles for age. Interestingly, either severe obesity (BMIZ ≥ 2.5) or BMIZ as a continuous variable was at least as good a predictor of higher IR as MetS in bivariate and multivariate analysis. Like MetS, severe obesity however, also had relatively low sensitivity and specificity as a predictor of higher IR in all age groups, and by itself would also lead to substantial numbers of false negatives and false positives for predicting IR. This goes to show that overweight children with IR defined as HOMA levels in the upper tertile may not be identified using the MetS criteria or severe obesity alone. Among individual MetS components, all were significantly associated with increasing HOMA in the 12–19 age group, whereas in the younger age groups only glucose and waist circumference showed statistically significant association, suggesting that the associations between IR, obesity and CVD risk factors likely increase with age.

Although MetS is believed to be closely related to IR, and both are independently associated with serious adverse outcomes among adults[34], recent studies in adults have shown that the widely accepted ATPIII criteria for MetS have low sensitivity for identifying adults with IR [36,37]. Several studies suggest that IR in children may also indicate increased and perhaps independent risk of subsequent CVD [25,38-40]. These studies showing the importance of IR did not suggest the MetS had to be present for additional CVD risk nor did they assess, to what extent MetS, a widely used clinical marker for IR and disease risk in overweight children, could be used to correctly identify children and adolescents with IR. As shown in this study using the modified ATP III criteria may fail to identify the potential increase in metabolic and cardiovascular disease risk in this younger population with IR but without MetS, at an age when risk factor intervention would be most beneficial.

Currently there are no widely accepted values to define IR in either normal or overweight children. Hence, markers of IR such as HOMA as described by Matthews and coworkers [41] have been used as a surrogate measure of IR in epidemiological studies to identify associated risk factors. HOMA as a measure of IR correlates with euglycemic clamp measures in men and women, younger and older adults, and obese and nonobese individuals [42-44]. It has recently been validated to correlate with insulin sensitivity indices obtained from the minimal model frequently sampled intravenous glucose tolerance test (FSIVGTT) in overweight prepubertal and pubertal children and adolescents [45]. Since no standard value for fasting insulin or HOMA has been validated as a predictor of CVD or diabetes, and these measures are known to vary by ethnicity and method of assay, most studies have used upper tertiles or quartiles for HOMA to identify insulin resistant individuals in the population being studied[34]. Higher IR thus defined has been found to be associated with significantly greater risk to develop type 2 diabetes, hypertension, CVD, and cancer [46-48] and similarly we chose to use this definition for our study. In this study the younger age group 3–8 years old had lower upper tertile HOMA values compared to the older children and adolescents, even though all groups had a similar prevalence of MetS by the modified ATPIII criteria. This may indicate that the clustering of metabolic risk factors in the younger children is more related to adiposity than IR and that the association with IR progresses over time.

Several reasons may account for the relatively low MetS sensitivity and specificity for identifying IR in this pediatric population. One is that in the ATP III definition all criteria are given equal importance in defining the syndrome such that impaired fasting glucose(IFG), the factor most closely associated with IR, need not be one of the ≥ 3 criteria for an individual to have MetS. If on the other hand IFG was a required criterion, the specificity for such a definition of MetS would be greater, while the sensitivity and positive predictive value would be far lower since very few (<4%) of the overweight children and adolescents in this sample had (IFG) ≥ 100 mg/dL. One reason for the relatively low proportion with elevated glucose may be that insulin resistance is generally associated with increased levels of insulin production. The ability of an insulin insensitive individual's beta cells to compensate for IR by producing extra quantities of insulin in response to a glucose load may decrease with age[49] and elevated glucose is a relatively late feature in the natural history of progression to type II diabetes.

Second unlike the WHO definition for MetS [50], the clustering of risk factors by ATP III definition may have multiple etiologies other than insulin resistance such as obesity, physical inactivity, and genetics[20]. Currently there is still no consensus on the diagnosis criteria of MetS in children and adults and whether MetS is one disease or a cluster of many obesity related risk factors with different underlying mechanisms. A recent review by Jones K [51]of metabolic syndrome in children raised similar questions as to the clinical value of recognizing MetS in children vs identifying the individual risk factors associated with obesity including those although not components of the MetS such as IR, LDL cholesterol and inflammatory markers, are nevertheless associated with CVD risk.

Other reasons for the low sensitivity of Mets criteria to identify IR are that, the deleterious effects of the prolonged presence of elevated insulin levels may take time to manifest themselves with regard to increase in triglycerides, blood pressure as well as circulating glucose levels as shown in this study where the associations of IR and metabolic risk factors were evident in the adolescents but not in the younger age groups. Alternately, as shown in a recent study by Lambert et al[52] for the pediatric population, some of these risk factors may be more reflective of adiposity than insulin resistance itself, leading to the modest specificity as well as sensitivity of MetS for IR. These observations of Lambert et al [52] and results of our study emphasize the fact that obesity in children represents heterogeneous metabolic and phenotypical expressions of insulin resistance and individuals with the same degree of obesity could have varying degrees of insulin resistance and metabolic aberrations

Our study has several limitations. The sample was drawn from participants in an obesity clinic situated with a predominantly African American and Hispanic catchments area. Thus our findings can only be generalized to overweight non-white youth. Our findings are nonetheless relevant given the higher prevalence of MetS found in this population compared to other studies [35] in patients of similar ethnic background, as well as higher rate of clinical complications of type II diabetes and CVD seen in adults of similar ethnic backgrounds.

In this study, we followed the example of others [46,47], in using upper tertile of HOMA as the measure of IR. It is possible that upper tertile may not be the appropriate threshold for IR in these overweight children. However, without data linking particular levels of HOMA with clinical outcomes, a specific clinical threshold is not yet available. In a study by Perichart- Perera et al, [53] IR defined by a HOMA value of 3.1 was present in 90% of obese Mexican children 9–12 years of age. It should be noted that the upper tertile values of HOMA in the overweight youth in our study are higher than this value and also higher then the upper tertile value in the general population observed by NHANES for children ≥ 12 years old which was 3.4 [54] and is thus more likely to indicate a potentially problematic level and improve the specificity of the analysis. In the study by Bueno G et al[55] mean HOMA levels were 5.6 for males and 5.5 for females diagnosed with MetS using similar Cook criteria in 10 year old Spanish children indicating significant IR even though their prevalence of MetS was lower compared to our study for the 9–11 age group. However when we used an even higher level threshold (upper quartile of HOMA) in sensitivity analyses, results were similar. This may reflect different degrees of insulin sensitivities in different ethnic groups.

It is also possible that the modified ATP III criteria for MetS that we used in this study may not be the most meaningful with regard to long-term clinical outcomes. The individual components and the specific cutoffs for children are not based on any prospective studies but have been defined by a consensus statement of an expert panel for adults. Since there is no universally accepted definition of MetS for children and the thresholds for adult ATP III criteria cannot be applied to a pediatric population given there are changes in the metabolic parameters as a function of age, we used modified ATP III adult criteria for children similar to that used by Cook et all which has been widely quoted and applied in different populations[17]. It is possible that using other MetS criteria such as proposed by De Ferranti et all[56] the prevalence of MetS in our study would be much higher as shown by others [55] and thus may increase the sensitivity of MetS to predict IR. We did not however test this definition as we have been routinely using the Cook definition in our clinic

In conclusion, the growing epidemic of childhood obesity, early onset of type II diabetes and clustering of cardiovascular risk factors has raised great concern. To date, this concern has led to increasing attention to the presence of MetS in children and adolescents. Even if MetS is an important marker or risk factor for CVD and type II diabetes in itself in a pediatric population, IR unaccompanied by MetS is also important.

The major benefit of defining MetS in children is to draw attention to risk factor clustering associated with obesity and insulin resistance. While the convergence of IR and MetS becomes evident over time these data suggest that in addition to screening for Mets, surrogate markers of insulin resistance such as fasting insulin, HOMA or if resources allow, glucose tolerance test, could be a useful addition to routine evaluations of overweight children in order to alert clinicians to potential increased risk in those with IR even without MetS. Further studies are needed to assess clinically relevant threshold values of insulin measures in overweight children and adolescents of different ethnicities that can predict the development of CVD, diabetes and other clinical syndromes in patients both with and without the metabolic syndrome.

## Abbreviations

National Cholesterol Education Program (NCEP)

Cardiovascular Disease(CVD)

Adult Treatment Panel (ATP)

Metabolic Syndrome (MetS)

Insulin Resistance (IR)

Homeostasis model assessment (HOMA)

Center for disease control (CDC)

Impaired Fasting glucose (IFG)

World Health Organization(WHO)

## Competing interests

The author(s) declare that they have no competing interests.

## Authors' contributions

All authors made a significant contribution in the conception, design and manuscript preparation.
